# Acute Kidney Injury Classification for Critically Ill Cirrhotic Patients: A Comparison of the KDIGO, AKIN, and RIFLE Classifications

**DOI:** 10.1038/srep23022

**Published:** 2016-03-17

**Authors:** Heng-Chih Pan, Yu-Shan Chien, Chang-Chyi Jenq, Ming-Hung Tsai, Pei-Chun Fan, Chih-Hsiang Chang, Ming-Yang Chang, Ya-Chung Tian, Ji-Tseng Fang, Chih-Wei Yang, Yung-Chang Chen

**Affiliations:** 1 Kidney Research Center, Department of Nephrology, Chang Gung Memorial Hospital, Taipei, Taiwan; 2 Division of Gastroenterology, Chang Gung Memorial Hospital, Taipei, Taiwan; 3 Chang Gung University College of Medicine, Taoyuan, Taiwan; 4 Department of Nephrology, Chang Gung Memorial Hospital, Keelung, Taiwan; 5 Department of Internal Medicine, Chang Gung Memorial Hospital, Taipei, Taiwan

## Abstract

Critically ill cirrhotic patients have high mortality rates, particularly when they present with acute kidney injury (AKI) on admission. The Kidney Disease: Improving Global Outcomes (KDIGO) group aimed to standardize the definition of AKI and recently published a new AKI classification. However, the efficacy of the KDIGO classification for predicting outcomes of critically ill cirrhotic patients is unclear. We prospectively enrolled 242 cirrhotic patients from a 10-bed specialized hepatogastroenterology intensive care unit (ICU) in a 2000-bed tertiary-care referral hospital. Demographic parameters and clinical variables on day 1 of admission were prospectively recorded. The overall in-hospital mortality rate was 62.8%. Liver diseases were usually attributed to hepatitis B viral infection (26.9%). The major cause of ICU admission was upper gastrointestinal bleeding (38.0%). Our result showed that the KDIGO classification had better discriminatory power than RIFLE and AKIN criteria in predicting in-hospital mortality. Cumulative survival rates at the 6-month after hospital discharge differed significantly between patients with and without AKI on ICU admission day. In summary, we identified that the outcome prediction performance of KDIGO classification is superior to that of AKIN or RIFLE classification in critically ill cirrhotic patients.

Acute kidney injury (AKI) is a common and serious complication in critically ill cirrhotic patients. The pathophysiological factors associated with AKI are also attributed to the dysfunction of other organs, indicating that AKI is often part of a multiple organ failure syndrome^1^,^2^. In the literature, the occurrence of AKI signifies a lower chance of survival in cirrhotic patients^3^,^4^,^5^,^6^,^7^,^8^.

To unify the definition of AKI, the Kidney Disease: Improving Global Outcomes (KDIGO) group classification was proposed based on the Risk, Injury, Failure, Loss of Kidney Function, and End-stage Kidney Disease (RIFLE) and the Acute Kidney Injury Network (AKIN) classifications in 2012^9^. An elevation of the serum creatinine (SCr) level exceeding 0.3 mg/dl within 48 h, or an increase in SCr to 1.5 times the baseline value, which is known or presumed to have occurred within 7 days before, or a urine volume of <0.5 ml·kg^−1^·h^−1^ for 6 h were defined as AKI. Like the AKIN criteria, KDIGO also classifies patients starting renal replacement therapy (RRT) as having stage 3 AKI, and it removes the threshold of a 0.5 mg/dl increment for SCr > 4 mg/dl in the criteria of stage 3 AKI. To date, the new AKI classification has been assessed in several investigations^10^,^11^,^12^,^13^,^14^. However, its application to critically ill cirrhotic patients and its prediction accuracy compared with the previous two classifications have not been thoroughly evaluated.

The objective of this prospective study was to determine the association between hospital mortality/short-term prognosis and the KDIGO classification in this homogeneous critically ill cirrhotic patient group. The AKIN and RIFLE classifications were also applied for comparison (Table 1).

## Results

### Patient characteristics

Between September 2012 and August 2014, 242 cirrhotic patients were enrolled at the specialized hepatogastroenterology ICU in our institution. The mean age of the patients was 58 years; 183 of them were male (75.7%) and 59 were female (24.3%). The overall in-hospital mortality rate was 62.8% (152 of 242), and the 6-month mortality was 77.7% (188 of 242). Table 2 lists the patient demographic data and the clinical characteristics of both survivors and nonsurvivors. Table 3 lists the causes of cirrhosis and the primary reasons for ICU admission. Liver diseases were usually attributed to hepatitis B viral infection (26.9%). The most frequent reason for ICU admission was upper gastrointestinal bleeding (38.0%).

### Comparison of AKI incidence according to the AKIN, RIFLE, and KDIGO classifications

The AKI severities determined by using the AKIN, RIFLE, and KDIGO classifications are listed in Table 4. The incidence rate of AKI was highest when using the KDIGO classification (67%). The KDIGO criteria identified 11 more patients with AKI than did the AKIN criteria; 1 of them was in stage I, and 10 were in stage III. The KDIGO criteria also identified 5 more patients with AKI than did the RIFLE criteria; 3 of them were in stage I and 2 were in stage III. When the overall patients were divided into the survivor and nonsurvivor groups, the AKI incidence of the survivor group determined by all the three classifications were almost the same and every single survivor was almost classified into the same AKI stage. However, in the nonsurvivor group, the KDIGO classification identified more patients with AKI than the other two classifications as mentioned above. The correlations between the scores of the AKIN, RIFLE, and KDIGO classifications on the first day of ICU admission are also listed in Table 5. The KDIGO classification showed positive correlations with the AKIN and RIFLE classifications in terms of the likelihood of in-hospital mortality (r > 0.25, *p* < 0.01). Concerning the correlation among the three criteria, the KDIGO criteria correlated better with the RIFLE criteria (0.988) than with the AKIN criteria (0.903). The correlation between the KDIGO and RIFLE criteria was also better than that between the RIFLE and AKIN criteria (0.900) (Table 5).

### Comparison of calibration and discrimination for predicting in-hospital mortality according to the AKIN, RIFLE, and KDIGO classifications

The in-hospital mortality rates of AKI patients defined by the AKIN, RIFLE, and KDIGO classifications were 77% (117 of 152), 80% (126 of 158), and 80% (130 of 163), respectively. The in-hospital mortality rate was significantly higher for patients with AKI than those without AKI, regardless of the applied criteria: AKIN (77% vs. 39%, *p* < 0.001), RIFLE (80% vs. 31%, *p* < 0.001), and KDIGO (80% vs. 28%, *p < *0.001) (Table 4). Table 6 shows the results of goodness-of-fit as measured by the Hosmer–Lemeshow χ^2^ statistic, denoting the predicted mortality risk and the predictive accuracy of the AKIN, RIFLE, and KDIGO classifications. A comparison between the discriminatory values of the three AKI classifications is also shown in Table 6. The AUROC analysis verified that the KDIGO classification had the best discriminatory power for predicting in-hospital mortality.

### Short-term prognosis of AKI and non-AKI patients defined according to the AKIN, RIFLE, and KDIGO classifications

The number of patients and the in-hospital mortality rate calculated according to the stratification data of the three AKI criteria are listed in Table 7. A progressive and significant increase in the mortality rate was observed to correlate with the increasing AKI stage defined according to the three AKI criteria. The increase of odds ratio between the increasing AKI stages is greatest in the KDIGO classification.

**Figure 1:**
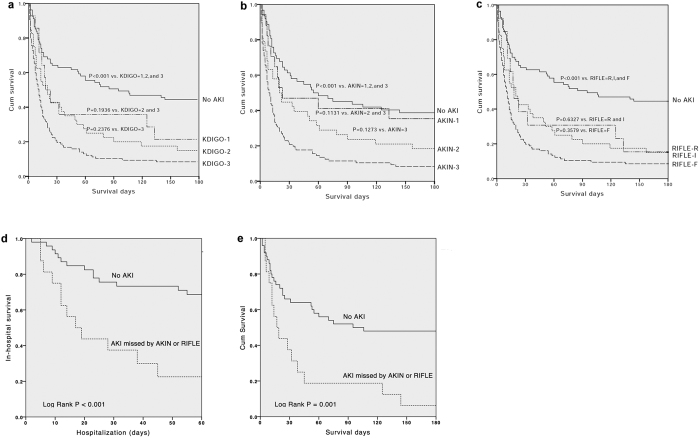
Survival Functions. Kaplan-Meier survival analysis for 242 critically ill cirrhotic patients according to the 3 AKI classifications on the first day of ICU admission. (**a**) Based on the KDIGO classification, the 180-day cumulative survival rates differed significantly for patients without AKI versus patients with KDIGO stages 1 to 3 (*p < 0.001*). The comparisons between patients with KDIGO stage 1 and those with KDIGO stages 2 to 3, and between patients with KDIGO stages 2 and those with KDIGO stage 3 have been depicted on the figure. (**b**) Based on the AKIN classification, the 180-day cumulative survival rates differed significantly for patients without AKI versus patients with AKIN stages 1 to 3, *p < 0.001*). The comparisons between patients with AKIN stage 1 and those with AKIN stages 2 to 3, and between patients with AKIN stages 2 and those with AKIN stage 3 have been depicted on the figure. (**c**) Based on the RIFLE classification, the 180-day cumulative survival rates differed significantly for patient without AKI versus patients with RIFLE-R, RIFLE-I, and RIFLE-F (*p < 0.001*). The comparisons between patients with RIFLE-R and those with RIFLE-I to F, and between patients with RIFLE-I and those with RIFLE-F have been depicted on the figure. (**d**) Patients with AKI diagnosed according to KDIGO but missed by AKIN or RIFLE classification had significantly lower hospital survival rate than patients without AKI (Log Rank P < 0.001). (**e**) Patients with AKI diagnosed according to KDIGO but missed by AKIN or RIFLE classification had significantly lower 180-day cumulative survival rate than patients without AKI (Log Rank P = 0.001). * Abbreviation: AKI, acute kidney injury; RIFLE, risk of renal failure, injury to kidney, failure of kidney function, loss of kidney function, and end-stage renal failure; AKIN, acute kidney injury network; KDIGO, kidney disease improving global outcomes.

Figure 1 shows that the 180-day cumulative survival rates differed significantly for patients with AKI and those without AKI defined by the three AKI criteria on the first day of ICU admission (*p* < 0.05).

## Discussion

This study included 242 cirrhotic patients with critical illnesses. The overall in-hospital mortality rate was 62.8%, which is well in keeping with that obtained in previous studies^5^,^15^,^16^. This investigation showed that the severity of AKI on the ICU admission day was associated with a significantly graded risk of death in critically ill cirrhotic patients, irrespective of which classification was used (Table 4). The analytical results also showed that the KDIGO classification was an excellent scoring system for predicting the outcome for critically ill cirrhotic patients (Table 6).

Several studies had compared the prediction accuracy of the KDIGO, AKIN, and RIFLE classifications. Most of the studies were performed retrospectively^10^,^11^,^12^,^13^. Luo X *et al*. had conducted a prospective investigation and showed that the KDIGO and AKIN classification had similar ability to predict mortality in critically ill patients, whereas the prediction accuracy of the RIFLE classification was inferior^14^. Our study focused on critically ill cirrhotic patients and a key aspect of this investigation is the definition of baseline SCr. For general population of patients without a previous SCr value before hospitalization, an alternative estimated baseline SCr value back-calculated by the Modification of Diet in Renal Disease (MDRD) formula have been widely adopted^12^,^14^. However, it is well known that creatinine-based equations, such as MDRD and Cockcroft-Gault formulas, are inaccurate in the estimation of glomerular filtration rate (GFR) in cirrhotic patients^17^,^18^. In this study, we used the last SCr value within the previous 3 months before hospitalization as the baseline SCr. For patients without an available SCr value before hospitalization, we followed the recommendations of the International Club of Ascites and used the first SCr value measured during hospitalization as the baseline SCr^18^,^19^. For taking account the specific feature of patients with cirrhosis, our study design might more precisely compare the prediction performance of the 3 AKI classifications in these patients.

The KDIGO classification reconciles the AKI definition of the RIFLE and AKIN classifications. In this investigation, the AKI incidence determined by using the KDIGO classification was higher than that obtained according to the RIFLE or AKIN classification. Compared with AKIN and RIFLE, the KDIGO classification identified 5% (11 of 242) and 2% (5 of 242) more patients fulfilling the AKI criteria (Table 4). Among the patients with AKI diagnosed according to KDIGO but missed with the AKIN or RIFLE classification, 82% (9 of 11) and 60% (3 of 5) of them died and accounted for 6% (9 of 152) and 2% (3 of 152) of the overall mortality, respectively. This AKI subgroup was also correlated to significant lower hospital and 180-day cumulative survival rates (Fig. 1). Tsien *et al*. observed that regardless of AKI episodes reversed or not, cirrhotic patients with AKI were more vulnerable to further renal dysfunction and to poor survival compared with those without AKI^20^. The greater sensitivity of the KDIGO classification might allow AKI episodes to be recognized earlier and make potential interventions possible.

In patients with chronic liver disease, the absolute level and relative change of SCr concentration are significantly lower than that in the general population^18^,^21^,^22^,^23^,^24^. Many studies have reported that even a minor fluctuation in SCr level appears to be strongly associated with adverse outcomes^13^,^25^,^26^. The percentage increase in SCr is even more obscure in patients with previous renal dysfunction^27^, which is particularly relevant among cirrhotic patients because of a high proportion of patients with preexisting impaired renal function on hospital admission^19^,^28^. The lack of capturing of small changes of SCr in RIFLE and considering the preadmission renal function in AKIN may explain their discriminative inferiority to the KDIGO classification (Table 6). Moreover, our data showed a progressive stepwise and significantly elevated in-hospital and 180-day mortality associated with increasing KDIGO stages (Table 7 and Fig. 1). On the basis of the study results, we strongly believe that the KDIGO classification is of great importance for standardizing the definition of AKI as well as facilitating advances in clinical practice and research.

Despite the encouraging results obtained in our study, several potential study limitations should also be considered. First, this study was conducted on patients from only one academic tertiary-care medical center, which limits the generalization of our findings. Our results may be unsuitable for direct extrapolation to other hospitals with different patient populations. Second, in our study, given that hepatitis B viral infection (27%) was the leading cause of liver cirrhosis, the use of our classification system may not be appropriate for patients in North America and in Europe where liver diseases are mostly attributed to hepatitis C viral infection and alcoholism. Third, sequential measurement of these scoring systems (e.g., daily or weekly) may reflect the dynamic aspects of clinical diseases, thus providing superior information on mortality risk. Fourth, the prognostic instruments were tested on patients already admitted to the specialized hepatogastroenterology ICU, rather than being used as a preadmission screening test, which may have skewed the measured results. Finally, the predictive accuracy of logistic regression models has its own limitations.

## Conclusion

This study showed the grave prognosis in critically ill cirrhotic patient with AKI. The analytical data demonstrated that the KDIGO classification is a better tool with superior prediction performance for short-term prognosis than the AKIN or RIFLE classification. We confirmed that the KDIGO classification is a great scoring system for risk stratification, and is capable of providing a more sensitive and standardized method for early AKI detection in critically ill cirrhotic patients.

## Materials and Methods

### Ethics statement

This clinical study was conducted in full compliance with the ethical principles of the Declaration of Helsinki and was consistent with Good Clinical Practice guidelines and the applicable local regulatory requirements. The local institutional review board of Chang Gung Memorial Hospital approved our study protocol (approval no. 98-3658A3). Patients who met the inclusion criteria were invited to participate in this study on their first day of admission to the intensive care unit (ICU). Trained physicians evaluated the patients’ mental status during the screening and proceeded to perform informed consent procedures. Written informed consent was obtained from all mentally competent patients or from the next-of-kin of compromised patients before their participation.

### Patient information and data collection

This study was conducted from September 2012 to August 2014, in a 10-bed specialized ICU (hepatogastroenterology ICU) at a 2000-bed tertiary-care referral hospital in Taiwan. In this study, we included 242 consecutive patients with hepatic cirrhosis requiring intensive monitoring and/or treatment unavailable elsewhere. The following patients were excluded: pediatric patients (age 18 years or below), patients or their next of kin who declined to be enrolled in the study, patients who stayed in the hospital for <24 h, patients who had a previous end-stage renal disease and were undergoing regular RRT, and patients who had undergone liver transplantation. For patients who were readmitted, we only recorded the clinical condition at the first admission to avoid double weighing the same patient.

Prospective data were collected, including demographic data, reason for admission to the ICU, immediate diagnosis, severity of the illness, serum and urine biochemical analysis, urine microscopy, urine output, duration of ICU and hospital stay, and treatment outcome. The Child–Pugh points, model for end-stage liver disease (MELD), Sequential Organ Failure Assessment (SOFA), Acute Physiology and Chronic Health Evaluation (APACHE) II and III scores determined on the first day of ICU admission, and related clinical data were also recorded. The primary study outcome was in-hospital mortality rate. Follow-up examinations were performed at 6 months after hospital discharge of the patients, by analyzing the chart records.

### Definitions

Cirrhosis was diagnosed on the basis of past medical history, the results of liver histology, or a combination of physical signs and symptoms and findings from biochemical analysis and ultrasonography. The severity of the liver disease on admission to the ICU was determined by using the Child–Pugh points and the MELD scoring system. The severity of the illness can also be assessed by using the APACHE II, APACHE III, and SOFA scores. The worst physiological and biochemical values determined on the first day of ICU admission were recorded.

The occurrence of AKI was determined by using the RIFLE, AKIN, and KDIGO classifications. We used the last SCr value within the previous 3 months before hospitalization as the baseline SCr in the RIFLE and KDIGO criteria. In patients without a previous SCr value, we used the first SCr value measured during hospitalization as the baseline SCr. For the AKIN criteria, the lowest SCr within 48 h before ICU admission was used as the baseline SCr. Both of the SCr and urine output criteria were considered in the three AKI classifications, and the criteria resulting in the worst possible classification were used. We applied a simple model for mortality, as follows: non-AKI (0 points); RIFLE-R, KDIGO stage 1, and AKIN stage 1 (1 point); RIFLE-I, KDIGO stage 2, and AKIN stage 2 (2 points); RIFLE-F, KDIGO stage 3, and AKIN stage 3 (3 points) on day 1 of ICU admission^29^,^30^.

Respiratory failure was defined as a respiratory rate of ≤5/min or ≥50/min, and/or the requirement for mechanical ventilation for ≥3 days, and/or fraction of inspired oxygen (FiO_2_) of >0.4, and/or a positive end-expiratory pressure of >5 cm H_2_O^31^,^32^,^33^. Sepsis was defined as systemic inflammatory response syndrome (SIRS) plus suspected or proven infection. According to the guidelines of the American College of Chest Physician/Society of Critical Care Medicine Consensus Conference, SIRS was defined as patients with more than one of the following clinical findings: body temperature, >38 °C or <36 °C; heart rate, >90 beats/min; hyperventilation evidenced by a respiratory rate of >20 cycles/min or a PaCO_2_ of <32 mm Hg; and a white blood cell count of >12,000 or <4000 cells/μL^34^.

### Statistical analysis

Continuous variables were summarized with means and standard derivations unless otherwise stated. Primary analysis compared hospital survivors with nonsurvivors. All variables were tested for normal distribution with the Kolmogorov–Smirnov test. Student’s t-test was applied to compare the means of continuous variables and normally distributed data; otherwise, the Mann–Whitney U-test was employed. This study used the χ^2^ test for trend to assess the categorical data associated with the RIFLE, AKIN, and KDIGO classifications. Correlations of paired-group variables were assessed by using linear regression and Pearson analysis.

Calibration was assessed by using the Hosmer–Lemeshow goodness-of-fit test (C-statistic) to compare the number of observed and predicted deaths in risk groups for the entire range of death probabilities. Discrimination was examined by using the area under the receiver operating characteristic curve (AUROC). To compare the areas under the two resulting AUROC curves, we used a nonparametric approach. Cumulative survival curves as a function of time were plotted by using the Kaplan–Meier approach and were compared by using the log rank test. All statistical tests were two-tailed, and a value of *p* < 0.05 was considered statistically significant. Data were analyzed with the Statistical Package for the Social Sciences software, version 19.0 for Windows (SPSS Inc., Chicago, IL, USA).

## Additional Information

**How to cite this article**: Pan, H.-C. *et al*. Acute Kidney Injury Classification for Critically Ill Cirrhotic Patients: A Comparison of the KDIGO, AKIN, and RIFLE Classifications. *Sci. Rep.* **6**, 23022; doi: 10.1038/srep23022 (2016).

## Supplementary Material

Supplementary Information

## Figures and Tables

**Table 1:** The definitions and classification for AKI.

	SCr criteria	UO Criteria
(A) RIFLE		
Definition	SCr changes over **1–7 days**, sustained for more than 24 hrs	UO < 0.5 ml/kg/h x 6 hrs
Risk	Increase in SCr ≥ 1.5 x baseline or decrease in GFR ≥ 25%	UO < 0.5 ml/kg/h x 6 hrs
Injury	Increase in SCr ≥ 2.0 x baseline or decrease in GFR ≥ 50%	UO < 0.5 ml/kg/h x 12 hrs
Failure	Increase in SCr ≥ 3.0 x baseline or an absolute serum creatinine ≥4.0 mg/dl with an acute rise of at least 0.5 mg/dl or or decrease in GFR ≥ 75%	UO < 0.5 ml/kg/h x 24 hrs or anuria x 12hrs
Loss	Complete loss of kidney function > 4 wks	
ESRD	End-stage renal disease (>3 mo)	
(B) AKIN		
Definition	Acute SCr changes occur within a **48 hrs** period during hospitalization	Same as RIFLE
Stage 1	**Increased in SCr of ≥0.3 mg/dl** or increase to 1.5–1.9 x baseline	
Stage 2	Increase in SCr to 2.0–2.9 x baseline	
Stage 3	Increase in SCr ≥ 3.0 x baseline or SCr ≥ 4.0 mg/dl with an acute rise of at least 0.5 mg/dl or **Initiation of RRT**	
(C) KDIGO		
Definition	SCr changes ≥ 1.5 x baseline to have occurred within the prior 7 days or a 0.3 mg/d lincrease in SCr must occur within a 48 hrs period	Same as RIFLE
Stage 1	Increased in SCr ≥ 1.5 x baseline or of 0.3 mg/dl	
Stage 2	Increase in SCr ≥ 2.0 x baseline	
Stage 3	Increase in SCr ≥ 3.0 x baseline or **increase in serum creatinine to ≥ 4.0 mg/dl** or initiation of RRT	

^*^Abbreviations: SCr, serum creatinine; UO, urine output; hrs, hours; wks, weeks; ESRD, end-stage renal disease; RRT, renal replacement therapy; RIFLE, risk of renal failure, injury to kidney, failure of kidney function, loss of kidney function, and end-stage renal failure; AKIN, acute kidney injury network; KDIGO, kidney disease improving global outcomes.

**Table 2:** Patients’ demographic data and clinical characteristics.

	All patients (n = 242)	Survivors (n = 90)	Non-survivors (n = 152)	p-value
Age (years)	58 ± 14	58 ± 14	58 ± 14	NS (0.775)
Gender (M/F)	183/59	72/18	111/41	NS (0.278)
Body weight (kg)	65.2 ± 12.7	65.3 ± 12.6	65.1 ± 12.8	NS (0.919)
Length of ICU stay (days)	8 ± 8	6 ± 6	9 ± 9	**0.002**
Length of hospital stay (days)	26 ± 24	29 ± 28	24 ± 22	NS (0.093)
MAP, ICU admission (mmHg)	72.3 ± 15.6	78.8 ± 14	68 ± 15	**<0.001**
Glasgow coma scale, ICU admission	9 ± 5	11 ± 5	8 ± 5	**<0.001**
Serum Creatinine, ICU first day (mg/dl)	2.6 ± 2.1	1.6 ± 1.3	3.2 ± 2.1	**<0.001**
Leukocytes, ICU first day (10^3/dl)	13.0 ± 7.9	10.8 ± 6.3	14.4 ± 8.5	**<0.001**
Hemoglobin, ICU first day (g/dl)	9.1 ± 2.1	9.1 ± 2.0	9.1 ± 2.2	NS (0.940)
Platelets, ICU first day (x10^9/l)	95 ± 74	90 ± 55	98 ± 83	NS (0.422)
Albumin, ICU first day (g/dl)	2.5 ± 0.6	2.6 ± 0.6	2.4 ± 0.5	**<0.001**
Sodium, ICU first day (mmol/l)	137 ± 10	138 ± 8	137 ± 10	NS (0.292)
Bilirubin, ICU first day (mg/dl)	10.2 ± 11.1	4.7 ± 6.5	13.9 ± 11.9	**<0.001**
Prothrombin time INR, ICU first day	2.3 ± 1.5	1.7 ± 0.5	2.7 ± 1.8	**<0.001**
AST, ICU first day	179 ± 501	95 ± 176	228 ± 613	**0.021**
ALT, ICU first day (units/l)	518 ± 1962	212 ± 412	704 ± 2450	**0.013**
DM (yes/no)	72/170	32/58	40/112	NS (0.147)
Hepatic encephalopathy (yes/no)	151/91	47/43	104/48	**0.014**
Previous ascites (yes/no)	186/56	68/22	118/34	NS (0.753)
Previous SBP (yes/no)	53/189	13/77	40/112	**0.036**
Previous EV bleeding (yes/no)	134/108	58/32	76/76	**0.033**
Previous peptic ulcer bleeding (yes/no)	85/157	29/61	56/96	NS (0.489)
Previous hepatoma (yes/no)	74/168	27/63	47/105	NS (1.000)
Previous renal failure (yes/no)	63/179	18/72	45/107	NS (0.129)
Respiratory failure, ICU first day (yes/no)	151/91	49/41	102/50	NS (0.055)
Sepsis, ICU admission (yes/no)	116/71	32/24	84/47	NS (0.412)
Child-Pugh points	11 ± 2	10 ± 2	12 ± 2	<0.001
MELD score	28 ± 12	19 ± 7	33 ± 10	**<0.001**
APACHE II	25 ± 9	20 ± 7	28 ± 9	**<0.001**
APACHE III	105 ± 40	78 ± 31	121 ± 35	**<0.001**
SOFA	11 ± 5	7 ± 3	13 ± 4	**<0.001**

Abbreviation: M, male; F, female; ICU, intensive care unit; MAP, mean arterial pressure; INR, international normalized ratio; AST, aspartate aminotransferase; ALT, alanine aminotransferase; DM, diabetes mellitus; SBP, spontaneous bacterial peritonitis; EV, esophageal varices; MELD, model for end-stage liver disease; APACHE, acute physiology and chronic health evaluation; SOFA, sequential organfailure assessment.

Values in bold are statistically significant (P-value < 0.05).

There were significant differences in length of ICU stay, mean arterial pressure, Glasgow coma scale, serum creatinine, albumin, bilirubin, AST, ALT, prothrombin time INR, leukocyte count, presence of hepatic encephalopathy, previous SBP, previous EV bleeding, liver and ICU prognostic scores.

**Table 3:** Causes of cirrhosis and reasons of ICU admission according to hospital survival.

	All patients (n = 242)	Survivors (n = 90)	Non-survivors (n = 152)	p-value
Causes of cirrhosis				
Alcoholic	54 (22.3%)	24 (26.7%)	30 (19.7%)	NS (0.263)
Hepatitis B	65 (26.9%)	19 (21.1%)	46 (30.3%)	NS (0.135)
Hepatitis C	52 (21.5%)	21 (23.3%)	31 (20.4%)	NS (0.629)
Alcoholic + Hepatitis B	30 (12.4%)	8 (8.9%)	22 (14.5%)	NS (0.231)
Alcoholic + Hepatitis C	7 (2.9%)	4 (4.4%)	3 (2.0%)	NS (0.429)
Hepatitis B + Hepatitis C	0 (0%)	0 (0%)	0 (0%)	–
Alcoholic + Hepatitis B + Hepatitis C	6 (2.5%)	1 (1.1%)	5 (3.3%)	NS (0.416)
Other causes^a^	28 (11.6%)	13 (14.4%)	15 (9.9%)	NS (0.303)
Primary ICU admission				
Severe UGI bleeding	92 (38.0%)	46 (51.1%)	46(30.3%)	**0.002**
Hepatic encephalopathy	60 (24.8%)	26 (28.9%)	34 (22.4%)	NS (0.283)
Respiratory failure	28 (11.6%)	6 (6.7%)	22 (14.5%)	NS (0.095)
Severe sepsis	47 (19.4%)	7 (7.8%)	40 (26.3%)	**<0.001**
HCC rupture	10 (4.1%)	4 (4.4%)	6 (3.9%)	NS (1.000)
Acute pancreatitis	2 (0.8%)	0 (0%)	2 (1.3%)	NS (0.531)
Acute renal failure	6 (2.5%)	1 (1.1%)	5 (3.3%)	NS (0.416)

Abbreviation: HCC, hepaocellular carcinoma; ICU, intensive care unit; UGI, upper gastrointestinal;

^a^ “Other causes” includes primary biliary cirrhosis, autoimmune hepatitis, andother unknown causes.

Values in bold are statistically significant (P-value < 0.05).

**Cause of cirrhosis:** none of the causes was independently associated with in-hospital mortality.

**Primary ICU admission reason:** sever UGI bleeding and severe sepsis were independently associated with in-hospital mortality.

**Table 4:** Survivors and non-survivors in different scoring system.

	All patients (n = 242)	Survivors (n = 90)	Non-survivors (n = 152)	p-value
AKIN				**<0.001**
No AKI	90 (37%)	55 (61%)	35 (23%)	**<0.001**
AKI	152 (63%)	35 (39%)	117 (77%)	**<0.001**
Stage I	15 (6%)	7 (8%)	8 (5%)	NS (0.286)
Stage II	40 (17%)	13 (14%)	27 (18%)	NS (0.436)
Stage III	97 (40%)	15 (17%)	82 (54%)	**<0.001**
RIFLE				**<0.001**
No AKI	84 (35%)	58 (64%)	26 (18%)	**<0.001**
AKI	158 (65%)	32 (36%)	126 (82%)	**<0.001**
Risk	13 (5%)	5 (6%)	8 (5%)	NS (1.000)
Injury	40 (17%)	12 (13%)	28 (18%)	NS (0.284)
Failure	105 (43%)	15 (17%)	90 (59%)	**<0.001**
KDIGO				**<0.001**
No AKI	79 (33%)	57 (63%)	22 (14%)	**<0.001**
AKI	163 (67%)	33 (37%)	130 (86%)	**<0.001**
Stage I	16 (7%)	6 (7%)	10 (7%)	NS (1.000)
Stage II	40 (17%)	12 (13%)	28 (18%)	NS (0.284)
Stage III	107 (44%)	15 (17%)	92 (61%)	**<0.001**

Abbreviation: AKI, acute kidney injury; RIFLE, risk of renal failure, injury to kidney, failure of kidney function, loss of kidney function, and end-stage renal failure; AKIN, acute kidney injury network; KDIGO, kidney disease improving global outcomes; NS, not significant

Values in bold are statistically significant (P-value < 0.05).

**Table 5:** Correlation between scoring systems on the first day of ICU admission (Spearman rank correlation coefficients: r).

Scores	AKIN	RIFLE
RIFLE	0.903**	–
KDIGO	0.903**	0.988**

Abbreviation: RIFLE, risk of renal failure, injury to kidney, failure of kidney function, loss of kidney function, and end-stage renal failure; AKIN, acute kidney injury network; KDIGO, kidney disease improving global outcomes

**p < 0.01.

**Table 6:** Comparison of calibration and discrimination of the AKI scores in predicting hospital mortality.

	Calibration			Discrimination		
	Hosmer-Lemeshow χ2	df	p	AUROC ± SE	95% CI	p
AKIN	1.739	2	0.419	0.741 ± 0.033	0.675–0.806	**<0.001**
RIFLE	2.302	2	0.316	0.774 ± 0.032	0.712–0.837	**<0.001**
KDIGO	2.473	2	0.290	0.781 ± 0.032	0.719–0.843	**<0.001**

Abbreviation: RIFLE, risk of renal failure, injury to kidney, failure of kidney function, loss of kidney function, and end-stage renal failure; AKIN, acute kidney injury network; KDIGO, kidney disease improving global outcomes; df, degree of freedom; AUROC, areas under the receiver operating characteristic curve; SE, standard error; CI, confidence intervals

Values in bold are statistically significant (P-value < 0.05).

**Table 7:** Odds Ratio of AKI stages in predicting hospital mortality according to AKIN, RIFLE, and KDIGO criteria.

Score	n	Hospital mortality (%)	Beta coefficient	Standard error	Odds rations (95% CI)	p
AKIN						
No AKI	90	39	–	–	1 (reference)	–
AKIN-1	15	53	0.097	0.950	1.101 (0.171–7.094)	0.919
AKIN-2	40	68	1.275	0.438	3.580 (1.518–8.442)	**0.004**
AKIN-3	97	85	2.188	0.386	8.922 (4.189–19.002)	**<0.001**
Constant	–	–	−0.502	0.264	0.605	0.057
RIFLE						
No AKI	84	31	–	–	1 (reference)	–
RIFLE-R	13	62	1.312	0.618	3.112 (1.105–12.470)	**0.044**
RIFLE-I	40	70	1.689	0.420	5.413 (2.377–12.327)	**<0.001**
RIFLE-F	105	86	2.644	0.367	14.075 (6.852–28.911)	**<0.001**
Constant	–	–	−0.842	0.239	0.431	**<0.001**
KDIGO						
No AKI	79	28	–	–	1 (reference)	–
KDIGO-1	16	63	1.153	0.592	3.167 (0.992–10.111)	**0.042**
KDIGO-2	40	70	1.712	0.422	5.542 (2.422–12.677)	**<0.001**
KDIGO-3	107	86	2.679	0.370	14.567 (7.057–30.070)	**<0.001**
Constant	–	–	−0.865	0.243	0.421	**<0.001**

Abbreviation: AKI, acute kidney injury, RIFLE, risk of renal failure, injury to kidney, failure of kidney function, loss of kidney function, and end-stage renal failure; AKIN, acute kidney injury network; KDIGO, kidney disease improving global outcomes.

Values in bold are statistically significant (P-value < 0.05).
