# Supplementary material for: Acute Kidney Injury Classification for Critically Ill Cirrhotic Patients: A Comparison of the KDIGO, AKIN, and RIFLE Classifications
Source: Sci Rep. 2016 Mar 17;6:23022. doi: 10.1038/srep23022 (PMC4794801; doi:10.1038/srep23022)
Supplement: Supplementary Information [file srep23022-s1.docx]

**Acute Kidney Injury Classification for Critically Ill Cirrhotic Patients: A Comparison of the KDIGO, AKIN, and RIFLE Classifications**

***Supplementary Information***

Heng-Chih Pan MD^1,4**^, Yu-Shan Chien^5**^, Chang-Chyi Jenq MD^1,3^, Ming-Hung Tsai MD^2,3^, Pei-Chun Fan MD^1^, Chih-Hsiang Chang MD^1^, Ming-Yang Chang MD, PHD^1,3^, Ya-Chung Tian MD, PHD^1,3^, Ji-Tseng Fang MD^1,3^, Chih-Wei Yang MD^1,3^, Yung-Chang Chen MD^1,3*^

^1^Kidney Research Center, Department of Nephrology, Chang Gung Memorial Hospital, Taipei,Taiwan,
^2^Division of Gastroenterology, Chang Gung Memorial Hospital, Taipei, Taiwan;
^3^Chang Gung University College of Medicine, Taoyuan, Taiwan;
^4^Department of Nephrology, Chang Gung Memorial Hospital, Keelung, Taiwan;
^5^Department of Internal Medicine, Chang Gung Memorial Hospital, Taipei, Taiwan

* The corresponding author

**These authors contributed equally to this work.

***Correspondence and reprint requests should be made to:***

Yung-Chang Chen, MD

Department of Nephrology

Chang Gung Memorial Hospital

199 Tung Hwa North Road, Taipei 105, Taiwan

Tel.: 886-3-3281200 ext 8181

Fax: 886-3-3282173

E-mail: [cyc2356@gmail.com](mailto:cyc2356@gmail.com)

Taiwan Consortium for Acute Kidney Injury and Renal Diseases (CAKs)

The members are listed according to their affiliation names in alphabetical order:

[Cardinal Tien Hospital]: Kuo-Cheng Lu, MD. [Chi-Mei Medical Center Liouying]: Jian-Jhong Wang, MD. [Chi-Mei Medical Center Yongkang]: Wei-Chih Kan, MD. [China Medical

University Hospital]: Chiu-Ching Huang, MD, Che-Yi Chou, MD, PhD., Ya-Fei Yang, MD. [Dalin Tzu-Chi Hospital]: Jen-Pi Tsai, MD. PhD. [Far Eastern Memorial Hospital]: Hung-Yuan Chen, MD.
[Hualien Tzu Chi Hospital]: Bang-Gee Hsu, MD, PhD.
 [International-Harvard Statistical Consulting Company]: Fu-Chang Hu, PhD.
[Kaohsiung Chang Gung Memorial Hospital]: Chien-Te Lee, MD, PhD., Jin-Bor Chen, MD., Chih-Hsiung Lee, MD, Wen-Chin Lee, MD, PhD., Lung-Chih Li, MD, PhD., Te-Chuan Chen, MD.
[Kaohsiung Medical University Chung-Ho Memorial Hospital]: Hung-Chun Chen, MD, PhD., Shang-Jyh Hwang, MD., Mei-Chuan Kuo, MD.
[Kaohsiung Municipal Ta-Tung Hospital]: Hugo You-Hsien Lin, MD.
[Keelung Chang Gung Memorial Hospital]: Chin-Chan Lee, MD., Chiao-Yin Sun, MD., Heng-Chih Pan, MD.
[Linkou Chang Gung Memorial Hospital]: Yung-Chang Chen, MD., Ming-Yang Chang, MD, PhD., Chang-Chyi Jenq, MD., Chan-Yu Lin, MD, PhD., Chih-Hsiang Chang, MD., Tsung-Yu Tsai, MD. [Lin-Shin Hospital]: Cheng-Min Chen, MD.
[Luodong Saint Mary’s Hospital]: Chih-Chung Shiao, MD.
[Mackay Memorial Hospital]: Chih-Jen Wu, MD, PhD., Cheng- Jua Lin, MD., Pei-Chen Wu, MD.
[Mackay Memorial Hospital Taitung Branch]: Feng-Chi Kuo, MD.
[Min-Sheng General Hospital]: Chih-Jen Weng, MD.
[National Health Research Institutes]: Li-Kwang Chen, PhD.
[National Taiwan University Hospital]: Kwan-Dun Wu, MD, PhD., Tzong-Shinn Chu. MD, PhD., Shuei-Liong Lin, MD, PhD., Vin-Cent Wu, MD, PhD., Chun-Fu Lai, MD, PhD. [National Taiwan University Hospital Bei-Hu Branch]: Tai-Shuan Lai, MD, PhD. [National Taiwan University Hospital Hsin-Chu Branch]: Wei-Shun Yang, MD.

[National Taiwan University Hospital Yun-Lin Branch]: Yung-Ming Chen, MD., Tao-Min Huang, MD.
[New Taipei City Hospital Sanchong Branch]: Wen-Ding Hsu, MD, MS.
[Shin-Kong Wo Ho-Su Memorial Hospital]: Jyh-Gang Leu, MD, PhD., Jui-Ting Chang MD. [Sin-Ren Hospital]: Hung-Hsiang Liou, MD.
[Taichung Veteran General Hospital]: Kuo-Hsiung Hsu, MD. Ming-Ju Wu, MD, PhD., Chun-Te Huang, MD.
[Taichung Veteran General Hospital Chiayi Branch]: Zi-hong You, MD.
[Taipei City Hospital Heping Branch]: Chao-Fu Chang, MD.

[Taipei Medical University Hospital]: Tzen-Wen Chen, MD. PhD., Hsi-Hsien Chen, MD. PhD., Fan-Chi Chang, MD. PhD., Yen-Chung Lin, MD., Mai-Szu Wu, MD., Chih-Chin Kao, MD.
[Taipei Tzu Chi Hospital]: Szu-Chun Hung, MD., Ko-Lin Kuo, MD, PhD., Che-Hsiung Wu, MD.
[Taipei Veterans General Hospital]: Der-Cherng Tarng, MD, PhD., Wu-Chang Yang, MD., Chih-Yu Yang, MD, PhD., Kuo-Hua Lee, MD.
[Taoyuan General Hospital, Ministry of Health and Welfare]: Wei-Jie Wang, MD, PhD., Sheng-Wen Ko, MD., Jui-Hsiang Lin, MD.
